# Machine learning automation

**DOI:** 10.1093/nsr/nwae288

**Published:** 2024-08-27

**Authors:** Zongben Xu, Zhi-Hua Zhou, Wenwu Zhu

**Affiliations:** School of Mathematics and Statistics, Xi'an Jiaotong University, China; National Key Laboratory for Novel Software Technology, Nanjing University, China; Department of Computer Science and Technology, Tsinghua University, China

With the exponential growth of big data and advancements in large-scale foundation model techniques, the field of machine learning has embarked on an unprecedented golden era. This period is characterized by significant innovations across various aspects of machine learning, including data exploitation, network architecture development, loss function settings and algorithmic innovation. These advancements have enabled machine learning applications to expand into diverse domains such as autonomous driving, artistic creation and AI-driven scientific research. Despite these rapid advancements, the increased complexity in configuring and deploying machine learning systems has significantly elevated the barriers to its effective applications, introducing formidable challenges for general users of machine learning algorithms.

Machine learning automation (MLA) emerges as a critical field of study in response to these challenges. It mainly aims to enhance adaptability and efficiency of machine learning, especially for dynamically changing open environments and diverse tasks, and is expected to facilitate the design of effective methodologies with minimal overhead. This area has attracted much research attention in recent years, driving forward the development of solutions that are both innovative and practical.

This special topic is dedicated to discussing the frontiers of MLA, with a special emphasis on elucidating recent developments for future explorations in this vital area. We have collected a total of eight papers in this special topic, including two Research Highlights, four Perspectives and two Reviews. For the two Research Highlights, Li and Cherukur [1] and Yao [2] introduced two recent MLA methodologies: open-environment machine learning (OpenML) [3] and the simulating learning methodology (SLeM) [4] for handling open environments and dynamically changing tasks, respectively. Li and Cherukur [1] introduced the main idea of OpenML and its intrinsic functions on robust artificial intelligence, and Yao [2] focused on introducing the task-transferable capability of SLeM beyond the current machine learning framework, mainly emphasizing the generalization capability at the data level.

For the four Perspectives, Xu *et al.* [5] comprehensively demonstrated the framework, theories, algorithms and applications of SLeM, and especially presented some MLA realization algorithms like data auto-selection, model auto-adjustment, loss auto-setting and algorithm auto-designing. They also introduced the potential applications of this framework for more practical datasets and tasks. Liu and Lin [6] presented an interesting study that utilizes bi-level optimization to uniformly formulate different kinds of MLA tasks from a unified optimization perspective, which is also hopeful to provide optimization tools for solving general MLA problems. Zhu [7] particularly suggested diffusion models for modeling the distribution of continuous-domain data and automatically generating new samples for certain learning tasks, emphasizing the training stability and strong model capacity of diffusion models. Huang [8] proposed a brief introduction to dynamic networks. Unlike traditional deep learning models that rely on fixed computational graphs and static parameters during inference, this approach can dynamically adjust its architecture and parameters based on varying inputs. This flexibility offers notable benefits in terms of accuracy, computational efficiency, adaptability and other key performance metrics.

For the two Reviews, Tang and Yao [9] provided an overview of recent research that utilizes a set of training examples from a specific category of optimization problems to derive shared knowledge, which can ease the burdensome algorithm configuration process for previously unseen problem instances. This review frames the discussion within the ‘Learn to Optimize’ paradigm, highlighting key concepts and significant challenges in this area. Wang and Zhu [10] focused on recent progress, applications, tools, benchmarks and future directions in neural architecture search, which primarily seeks to systematically explore complex architecture spaces to identify optimal neural configurations with minimal manual intervention.

Although this special topic cannot comprehensively cover all approaches in the research area of MLA, it highlights some of the most representative recent developments in this field. We hope that these contributions will inspire further advancements and encourage more researchers to explore this area, thereby driving progress in MLA research.
